# SARS-CoV-2 viral load in sputum correlates with risk of COVID-19 progression

**DOI:** 10.1186/s13054-020-02893-8

**Published:** 2020-04-23

**Authors:** Xia Yu, Shanshan Sun, Yu Shi, Hao Wang, Ruihong Zhao, Jifang Sheng

**Affiliations:** State Key Laboratory for Diagnosis and Treatment of Infectious Diseases, National Clinical Research Center for Infectious Diseases, Collaborative Innovation Center for Diagnosis and Treatment of Infectious Diseases, The First Affiliated Hospital, College of Medicine, Zhejiang University, Qingchun Road, No79, Hangzhou, 310003 China

**Keywords:** SARS-CoV-2, Viral load, Disease severity, Disease progression

The pandemic of coronavirus diseases 2019 (COVID-19) imposes a heavy burden on medical resources [1]. Whether there is correlation between viral load and disease severity has not been clarified. In the study, we retrospectively collected the virological data, as well as demographic, epidemiological clinical information of 92 patients with confirmed COVID-19 in a single hospital in Zhejiang Province, China. We compared the baseline viral loads between severe patients and those mild to moderate at admission and also between those developing severe disease during hospitalization and those not.

We studied 92 patients with confirmed COVID-19 who were admitted from January 19, 2020, to March 19, 2020, in the First Affiliated Hospital of Zhejiang University. The sputum specimens were collected from the lower respiratory tract of each patient at admission and the levels of viral nuclei acid were determined by a real-time PCR (RT-PCR) approach and indicated by the cycle threshold (Ct) values of RT-PCR assays [2]. Other demographic, epidemiological and clinical information were collected and inputted into a pre-designated electronic data collection form. All patients followed up to March 15, 2020. All the statistical analyses were performed using GraphPad Prism 5 (GraphPad Software Inc.; San Diego, CA, USA) and SPSS 20.0 (SPSS Inc.; Chicago, IL, USA).

Of the 92 patients, 30 were severe on admission. Of the other 62 mild-moderate cases at admission, 11 cases became severe during hospitalization. The demographic, epidemiological and clinical information was shown in Table 1. All patients were tested for SARS-CoV-2 nucleic acid on sputum specimens from the lower respiratory tract at admission. As shown in Fig. 1a, severe patients had significantly lower Ct values than mild-moderate cases at admission (25 vs. 28, *p* = 0.017), suggesting a higher viral load in the lower respiratory tract. Furthermore, a higher viral load was observed in sputum specimens from patients who became severe during the hospitalization than those did not (24 vs. 29, *p* = 0.008). As shown in Fig. 1b, the Ct values of RT-PCR assays negatively correlated with the probability of progression to severe type in all the patients representing mild-to-moderate at admission.

**Table 1:** Demographic, comorbidities, epidemiological characteristics, and clinical and laboratory findings of patients with confirmed COVID-19 at admission

Variables	Total (*n* = 92)	Mild-moderate at admission				Severe at admission (*n* = 30)	*P* value*
		Persistent mild-moderate during hospitalization (*n* = 51)	Mild-moderate to severe during hospitalization (*n* = 11)	*P* value^***#***^	Total (*n* = 62)		
**Demographic data**							
Age (years)	55 ± 16	49 ± 13	59 ± 17	0.032	51 ± 15	63 ± 16	0.001
Sex							
Male	57 (62%)	26 (51%)	8 (72.7%)		34 (54.8%)	23 (76.7%)	
Female	35 (38%)	25 (49%)	3 (27.3%)	0.189	28 (45.2%)	7 (23.3%)	0.043
Occupation							
Agricultural worker	45 (48.9%)	25 (49%)	7 (63.6%)		32 (51.6%)	13 (43.3%)	
Self-employed	21 (22.8%)	15 (29.4%)	2 (18.7%)		17 (27.4%)	4 (13.3%)	
Employee	8 (8.7%)	5 (9.8%)	0 (0%)		5 (8.1%)	3 (10%)	
Retired	17 (18.5%)	5 (9.8%)	2 (18.2%)		7 (11.3%)	10 (33.3%)	
Students	1 (1.1%)	1 (2%)	0 (0%)	0.669	1 (1.6%)	0 (0%)	0.082
Smoking history							
Yes	16 (17.4%)	7 (13.7%)	3 (27.3%)		17 (27.4%)	6 (20%)	
No	76 (82.6%)	44 (86.3%)	8 (72.7%)	0.268	45 (72.6%)	24 (80%)	0.441
**Comorbidities**							
Hypertension	33 (35.9%)	10 (19.6%)	7 (63.6%)	0.003	17 (27.4%)	16 (53.3%)	0.016
Diabetes	9 (9.8%)	1 (2%)	2 (18.2%)	0.024	3 (4.8%)	6 (20%)	0.022
Cardiovascular disease	8 (8.7%)	2 (3.9%)	1 (9.1%)	0.472	3 (4.8%)	5 (16.7%)	0.060
Chronic liver diseases	4 (4.3%)	2 (3.9%)	1 (9.1%)	0.472	3 (4.8%)	1 (3.3%)	0.741
Chronic renal diseases	3 (3.3%)	0 (0%)	1 (9.1%)	0.031	1 (1.6%)	2 (6.7%)	0.203
Others	6 (6.5%)	0 (0%)	2 (18.2%)	0.002	2 (3.2%)	4 (13.3%)	0.067
**Epidemiological characteristics**							
Exposure to confirmed cases	46 (50%)	30 (58.8%)	5 (45.5%)	0.421	35 (56.5%)	11 (36.7%)	0.077
Family cluster	27 (29.3%)	15 (29.4%)	4 (36.4%)	0.653	19 (30.6%)	8 (26.7%)	0.696
Recent travel or residence to/in epidemic area	25 (27.2%)	11 (21.6%)	4 (36.4%)	0.303	15 (24.2%)	10 (33.3%)	0.358
**Signs and symptoms**							
Fever	84 (91.3%)	45 (88.2%)	11 (100%)	0.235	56 (90.3%)	28 (93.3%)	0.633
Cough	58 (63%)	32 (62.7%)	7 (63.6%)	0.956	39 (62.9%)	13 (43.3%)	0.968
Fatigue	6 (6.5%)	1 (2%)	2 (18.2%)	0.024	3 (4.8%)	3 (10%)	0.350
Diarrhea	7 (7.6%)	1 (2%)	1 (9.1%)	0.229	2 (3.2%)	5 (16.7%)	0.023
Nausea and vomiting	4 (4.3%)	3 (5.9%)	1 (9.1%)	0.697	4 (6.5%)	0 (0%)	0.157
Shortness of breath	25 (27.2%)	2 (3.9%)	4 (36.4%)	0.001	6 (9.7%)	19 (63.3%)	< 0.001
Time to admission	3 (4)	4 (3)	1 (4)	0.011	4 (4)	1 (4)	0.211
Time to confirmed diagnosis	5 (5)	5 (4)	4 (4)	0.160	5 (4)	3 (6)	0.239
**Laboratory parameters**							
WBC	6.5 (5.9)	5.2 (4.1)	7.5 ± 3.4	0.188	5.4 (4.5)	10.8 ± 5.6	< 0.001
Lymphocyte	0.8 (0.6)	0.97 ± 0.47	0.7 (0.4)	0.147	0.9 (0.7)	0.5 (0.5)	0.001
Platelet	191 (76)	193 (83)	170 ± 56	0.159	192 (84)	191 ± 45	0.851
CRP	27 (37)	13 (27)	37 ± 27	0.036	16 (30)	39 (29)	< 0.001
ALT	23 (22)	23 (24)	17 (15)	0.338	22 (23)	23 (16)	0.723
AST	22 (16)	21 (12)	21 (18)	1.000	21 (12)	26 (23)	0.236
Cr	75 (25)	71 ± 26	84 (39)	0.054	73 (28)	84 (33)	0.019
INR	0.98 (0.09)	0.97 ± 0.08	0.97 (0.04)	0.507	0.97 ± 0.06	1.01 ± 0.09	0.050
Bilirubin	10.8 (6.0)	12.2 (5.0)	10.0 (6.0)	0.912	10.6 (5.0)	12.6 (9.0)	0.097
LDH	281 ± 105	227 (103)	279 ± 101	0.376	229 (113)	339 (121)	< 0.001
CK	70 (76)	63 (61)	76 (60)	0.495	64 (58)	97 (172)	0.011
Urea nitrogen	5.3 (3.7)	4.4 (1.7)	6.8 (6.9)	< 0.001	4.6 (2.2)	7.7 (4.2)	< 0.001
CT scan							
Normal	3 (3.3%)	3 (5.9%)	0 (0%)		3 (4.8%)	0 (0%)	
Local lesion	5 (5.4%)	4 (7.8%)	0 (0%)		4 (6.5%)	1 (3.3%)	
Multi-lesions	84 (91.3%)	44 (86.3%)	11 (100%)	1.000	55 (88.7%)	29 (96.7%)	1.000
ICU admission	27 (29.3%)	0 (0%)	8 (72.7%)	< 0.001	8 (12.9%)	19 (63.3%)	< 0.001

Data are expressed as number (percent), mean ± standard deviation (SD), or median (IQR)

^#^*P* values comparing data between patients becoming severe and those who did not during hospitalization by the Mann-Whitney *U* test, chi-squared test, or Fisher’s exact test

*P values comparing data between mild-moderate patients and severe patients at admission

**Fig. 1:**
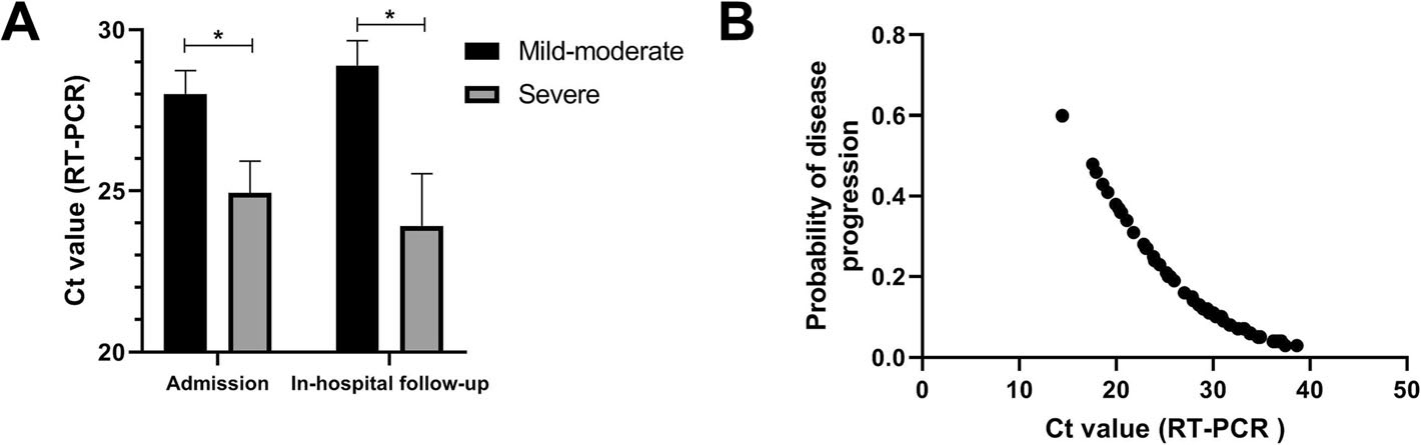
**a** Comparison of baseline sputum viral load between severe and mild-to-moderate patients at admission or between those becoming severe and those did not during the hospitalization. **b** Relationship between the estimated probability of disease progression during the hospitalization and baseline sputum viral load. Viral load is indicated by the Ct value of RT-PCR assay. The asterisk indicates a *P* value < 0.05

We found that the viral load of the sputum specimen in the lower respiratory tract tested at baseline is closely related to the severity of COVID-19. More importantly, patients with a higher baseline viral load are more likely to become severe. This finding apparently justifies the concept that early antiviral treatment, if effective, would reduce the risk of progression and thereby the mortality, which has been demonstrated in influenza [3]. In our study, sputum specimens were used, instead of nasopharyngeal and oropharyngeal swabs because it has been shown that samples from lower respiratory tract generally contain a higher level of viral load than nasopharyngeal and oropharyngeal swabs [4] and acquiring swabs is uncomfortable for patients.

In summary, we found a positive association between sputum viral load and disease severity as well as risk of progression.

## Data Availability

The datasets and materials used and/or analyzed during the current study are available from the corresponding author on reasonable request.
